# Toxoplasmosis vaccines: what we have and where to go?

**DOI:** 10.1038/s41541-022-00563-0

**Published:** 2022-10-31

**Authors:** Yizhuo Zhang, Dan Li, Shaohong Lu, Bin Zheng

**Affiliations:** 1 Institute of Parasitic Diseases, School of Basic Medicine and Forensics, Hangzhou Medical College, Hangzhou, China; 2 Engineering Research Center of Novel Vaccine of Zhejiang Province, Hangzhou Medical College, Hangzhou, China; 3 Key Laboratory of Bio-tech Vaccine of Zhejiang Province, Hangzhou Medical College, Hangzhou, China

**Keywords:** Infectious diseases, Vaccines

## Abstract

Despite recent major advances in developing effective vaccines against toxoplasmosis, finding new protective vaccination strategies remains a challenging and elusive goal as it is critical to prevent the disease. Over the past few years, various experimental approaches have shown that developing an effective vaccine against *T. gondii* is achievable. However, more remains unknown due to its complicated life cycle, difficulties in clinical translation, and lack of a standardized platform. This minireview summarizes the recent advances in the development of *T. gondii* vaccines and the main obstacles to developing a safe, effective and durable *T. gondii* vaccine. The successes and failures in developing and testing vaccine candidates for the *T. gondii* vaccine are also discussed, which may facilitate the future development of *T. gondii* vaccines.

## Introduction

*Toxoplasma gondii (T. gondii)* is an obligate intracellular protozoan from the Phylum Apicomplexa that can infect all warm-blooded animals. Infections in healthy individuals are generally asymptomatic, while they can be severe or even life-threatening among immune-compromised patients. The vertical transmission of *T. gondii* from mother to child is particularly concerning. In addition, toxoplasmosis has severe effects on animals. It causes certain economic losses to animal husbandry and adversely affects food safety. Chemotherapies are commonly used for the treatment of toxoplasmosis. The combination of pyrimethamine and sulfadiazine is the gold standard in treating toxoplasmosis. However, the treatment success rate remains low, as it can only kill tachyzoites but not bradyzoites. Furthermore, the treatment also causes significant side effects^1^. It may induce a folate deficiency state, which is probably responsible for hematological side effects and embryopathies. It is also associated with rare severe reactions that may be fatal, including agranulocytosis, Stevens-Johnson syndrome, toxic epidermal necrolysis, and hepatic necrosis. In the case of *T. gondii*, vaccination against toxoplasmosis could be an effective and appropriate medical prevention, especially for specific populations (pregnant women and HIV patients)^2^. However, vaccines against *T. gondii* infection in humans are still under development, despite vaccines for domestic animals and livestock being commercially available. A live attenuated vaccine (Toxovax®, MSD, New Zealand) consisting of a modified *T. gondii* strain (S48) is approved in some regions (Europe and New Zealand) to reduce losses to the sheep industry due to congenital toxoplasmosis^3^. The results indicate that the *T. gondii* vaccine can be successfully developed and commercialized for human immunization. However, developing an ideal vaccine remains a significant challenge due to the complexities of the *T. gondii* genome, life cycle, and strain diversity.

## Anti-*T. gondii* vaccines

**Fig. 1:**
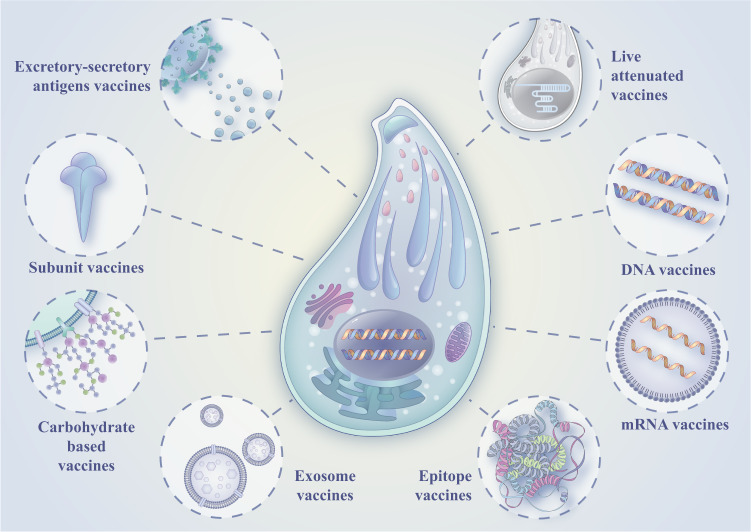
Anti-*T. gondii* vaccines.

Since the middle of the last century, the research on *T. gondii* vaccines has gone through different stages, mainly including inactivated vaccines, excretory-secretory antigen vaccines, live attenuated vaccines, subunit vaccines, DNA vaccines, epitope vaccines, and mRNA vaccines (Fig. 1). Although these vaccines can play a particular role in preventing and treating toxoplasmosis, there are also some problems.

### Vaccines based on excretory-secretory antigens

Excretory-secretory antigens (ESA) produced by tachyzoites account for most circulating antigens in the serum and cerebrospinal fluid of the host. ESA immunization can improve animal survival rates by reducing parasitemia of highly virulent strains and controlling infections^4^. Subcutaneous injection of ESA reduced the formation of tissue cysts in pigs after *T. gondii* infection compared to the control group^5^. However, ESA immunization does not provide complete immune protection and cannot overcome the complex strains and life cycle of *T. gondii*. An adjuvant mixture of propranolol (PRP), a β-adrenergic receptor antagonist, and aluminium (alum) was used for immunization, significantly improving the protective effect of ESA and extending the survival time of mice^6^.

### Live attenuated vaccines

Gamma irradiation, chemical treatment and multiple passages are commonly used to generate an attenuated *T. gondii* line incapable of completing its life cycle with reduced virulence. At the same time, the strain’s antigenicity is retained to elicit an immune response in the host, allowing it to produce memory cells and prevent reinfection. Currently, only one live attenuated vaccine, Toxovax®, is commercially available to reduce the damage caused to the sheep industry by congenital toxoplasmosis. The vaccine consists of a modified strain of *T. gondii* (S48 stain), generated upon years of repeated passages in mice. The vaccine strain has lost the ability to form oocyst^7^. T-263, a chemical-induced *T*. *gondii* mutant, is also immunogenic but incapable of forming oocysts in cats. It has been reported that cats fed live bradyzoites of T-263 do not excrete oocysts after the challenge with oocyst-producing strains^8^.

With the rapid development of gene-editing technology, CRISPR/Cas9 has become a revolutionary, powerful and precise technology for genome editing in many organisms. The CRISPR/Cas9 system is a powerful tool to generate a specific loss-of-function phenotype by gene knockout. It has become possible to produce live attenuated vaccines by controlling and changing substances required for *T. gondii* growth and reproduction. With promising results, many gene knockouts and attenuated live vaccines of *T*. *gondii* have been studied using the CRISPR/cas9 system^9^. Chandra et al. showed that the Δ*HAP2* parasites could not complete fertilization and meiosis and could only produce a few abnormal oocysts. Inoculation of cats with Δ*HAP2* parasites completely prevented wild-type *T. gondii* oocysts from shedding^10^. Besides, both Ca^2^^+^-dependent protein kinase 2 knocked out (Δ*CDPK2)* strain of *T. gondii* and the adenylosuccinate lyase knocked out(Δ*ADSL*) strain of *T. gondii* Live attenuated vaccines induce anti-*T. gondii* humoral and cellular immune responses and have 100% immune protection in their experiment^11,12^.

Oral live attenuated vaccine mimics the natural infection state of *T. gondii* and induces host cellular and humoral immunity against *T. gondii* without causing disease. Current *T. gondii* vaccine research indicates the live attenuated vaccine as the most effective. Nevertheless, live attenuated vaccines also have drawbacks, such as short shelf life, safety issues for handling personnel, ethical reasons, and safety issues, making them unsuitable for humans. Furthermore, because of their unknown genetic background, attenuated vaccines may be counterproductive by reverting to their virulent wild-type and causing the diseases they are designed to prevent^3^.

### Subunit vaccines

Subunit vaccines are created using only the parts of *T*. *gondii* required by the immune system. So far, many *T. gondii* subunit vaccines have been systematically studied. Recombinant heat shock protein 70 (rTgHSP70)-immunized mice induced high and sustained nitric oxide (NO) production in peritoneal macrophages, and rTgHSP70 immunity also enhanced the expression of iNOS in the brain and reduced the occurrence of cerebral cysts. rTgHSP70 immunization reduced parasite numbers^13^. The subunit vaccines with multiple epitope designs are more valuable for research. Onile et al. used immune informatics tools to design multi-epitope subunit vaccines with different T-cell and B-cell epitopes to fight toxoplasmosis^14^. Two recombinant proteins, recombinant rhoptry protein 18 (rROP18) and recombinant calcium-dependent protein kinase 6 (rCDPK6), combined with Poly (lactide-co-glycolide) (PLG), a biodegradable and biocompatible polymer, prolong the protein release period, lower protein degradation and induce a long-lasting immune response. The brain cysts load of immunized mice was significantly lower than that of the control group^15,16^.

The main disadvantage of subunit vaccines is that they provide less immune protection and usually require carrier or adjuvant delivery. However, using nanoparticles as carriers in the design of candidate subunit vaccines can significantly improve immune protection against *T. gondii*^17^.

### Genetically engineered vaccines

#### DNA vaccines

Most DNA vaccines are based on parasite virulence-related proteins. These proteins include rhoptry proteins (ROP), dense granular proteins (GRA), microsomal proteins (MIC), and surface antigens (SAG)^18^. ROP proteins are involved in cell invasion and the formation of parasitic vacuoles. They are critical for *T*. *gondii* survival in host cells. DNA vaccination of ROP1, ROP8, ROP13, ROP16, ROP18, and ROP54 antigens induces dominant Th1-mediated immunity, corresponding to the production of cytokines such as IL-22, IL-2, IL-5, and IFN-γ, extending survival time of immunization mice^19–24^. For example, ROP16 is a key virulence factor in the pathogenesis of *T. gondii* because it can directly subvert the signal transducer and activator of the transcription 3/6 (STAT3/6) signal and target the host cell nucleus^22^. ROP16-based DNA vaccines can significantly enhance cytokine IFN-γ, IL-2, and IL-4 production for acute infections and increase survival by 13–27 days^22^. The GRAs modify the parasitophorous vacuole (PV) and parasitophorous vacuole membrane (PVM) for maintaining intracellular parasitism in host cells and are involved in parasite survival, virulence and replication. Among GRAs, There are many studies on GRA-24, GRA-7, GRA-4, GRA-2, and GRA-1. These antigens induce a mixed Th1/Th2 -mediated immunity, corresponding to the production of cytokines such as IL-4, IL-10, IL-12, IFN-γ, and TNF-α. For example, GRA-24 induced high levels of mixed Th1/Th2 cytokines 6 weeks after immunization, and the survival times were prolonged significantly (24.6 ± 5.5 days)^25–27^. The MICs are exposed on the tachyzoite surface and bind to host cell surface receptors to promote tachyzoite adhesion and subsequent host cell invasion. Among MICs, MIC2, MIC3, MIC4, MIC8, MIC11, and MIC13 antigens have been evaluated. These can induce the humoral and Th1-type immune responses, significantly enhance IFN-γ, IL-12, and IL-2 production, and higher survival time. The MIC8 DNA vaccine induces strong humoral and cellular immune responses and produces cytokines such as IL-15 and IL-21, which were protective against *T. gondii* challenge^28,29^. SAGs are mainly involved in the recognition and adhesion of *T. gondii* to host cells and the early invasion process. SAG1 (P30), SAG2 (P22), and SAG3 (P43) are the major SAGs. The SAG1 was sufficiently immunogenic to elicit cellular and humoral responses that led to a high degree of protection of animals against *T. gondii* infection. However, this vaccine has the limitation of being a tachyzoites stage-specific antigen^30^.

Although many DNA vaccines are inexpensive, easy to administer and induce a strong immune response at low doses, many obstacles still exist. For example, immunity is often weak in large animals, requiring 1000 times as much DNA vaccine as in small animals to be effective^31^. In addition, potential genome integration of plasmids may activate oncoproteins and produce antibodies in the DNA vaccine itself^32^.

#### Epitope vaccines

Because *T. gondii* has a complex life cycle with different epitopes at different stages, multiple epitopes are frequently employed to design vaccines. The protective effect of the polyvalent epitope vaccine was better than that of the single epitope vaccine. Mcleod’s group has made significant progress in identifying vaccine prototypes entering clinical trials. Using HLA transgenic mice as animal models, they combined epitopes of *T. gondii* at various stages with self-assembling protein nanoparticles (SAPNs) and induced mice with strong CD8^+^ T and CD4^+^ T-cell responses combined with an adjuvant, thus prolonging the survival time of mice^33–36^. Furthermore, numerous protein epitope vaccines have been thoroughly investigated, making significant progress^37^. Roozbehani’s group also successfully used SAPNs as scaffolds/platforms, which combined with peptide epitopes derived from SAG1, SAG2C, GRA6, GRA5, and so on for vaccine delivery. In addition, mice vaccinated with the multi-epitope-based vaccine generated more significant Th1 immune responses and had increased survival rates, specific antibody titers, and IFN-γ and IL-2 levels than controls^38^.

Compared to other types of vaccines, epitope-oriented vaccines have a lower risk of biohazard and the ability to design and optimize epitope structures, enhancing the potential of vaccines and inducing stronger immunity. Due to the lack of secondary and tertiary structure of the natural protein, epitope vaccines have small molecules, poor immunogenicity and a short half-life, which is less potent in stimulating a protective immune response and may cause immune tolerance. Therefore, improving the affinity of vaccine-induced antibodies and increasing the immunogenicity and stability of epitope vaccines has recently been a research hotspot.

### Novel vaccines

#### mRNA vaccines

The mRNA vaccines introduce the mRNA coding *T. gondii* antigen target into the host through a specific delivery system, where the protein is expressed in the host and stimulates the host to produce a specific immunological response so that the host can obtain protective immunity. Experiments confirmed that an mRNA vaccine based on *T. gondii* nucleoside triphosphate hydrolase-II (TgNTPase-II), administered to mice for immunization by synthetic lipid nanoparticles (LNP), can stimulate robust humoral and cellular immunity, resulting in high antibodies and IFN-γ^39^. The results showed that the cyst number in the brain decreased significantly in the experimental group, and the survival time was also significantly extended. Combining mRNA and LNP will contribute to developing safe and long-acting toxoplasmosis vaccines^39^.

mRNA vaccines do not have to generate a large quantity of live attenuated *T. gondii* parasites, which may also revert to their virulent wild-type. In contrast to epitope vaccines and subunit vaccines, mRNA vaccines can overcome the challenges of inappropriate folding during the expression of recombinant proteins in vitro. Moreover, mRNA vaccines are more suitable for preventing outbreaks of pathogens than DNA vaccines. mRNA vaccines are relatively safe as they are confined to the cytoplasm, theoretically avoiding the risk of gene recombination and reducing malignant cell conversion. Further, despite an earlier start for research and development, there is still no DNA vaccine approved for human use. In contrast, many mRNA vaccines are already licensed (for example, COVID-19 mRNA vaccine). mRNA vaccine access is through the process of enzyme in vitro transcription production, which does not rely on cell amplification, so the antigen of cell culture, extraction, and purification process are saved, and the production time is shortened. So the time required to design and test new mRNA vaccines is also very short, with significant advantages against outbreaks such as influenza and COVID-19^40,41^. However, mRNA vaccines have a short intracellular half-life and easily degrade in vivo and during storage. As a result, delivery via substances such as liposomes is typically required^42^.

#### Carbohydrate-based vaccines

Carbohydrates on the surface of *T. gondii* are crucial for the infection of animal and human hosts. Its proteins are tagged with carbohydrates to stabilize and transport them, critical to completing the *T. gondii* life cycle. Carbohydrate antigens are recognized by the host’s immune system, eliciting carbohydrate-specific antibodies, making carbohydrates an attractive target for vaccine development^43^. TLR-2 and TLR-4 can recognize *T. gondii* GPI, inducing an inflammatory response^44^. However, the immune response induced by carbohydrate vaccines was insufficient to resist the attack of lethal doses of *T. gondii* tachyzoite^44^.

Carbohydrate vaccines currently leave much to be desired. Carbohydrates are often less immunogenic than proteins, and less likely to stimulate high-affinity antibodies. Moreover, the structure of carbohydrates tends to be similar to that of the host, which may lead to autoimmunity^45^.

#### Exosome vaccines

Exosomes can migrate through the intestinal basement membrane by directly stimulating T cells or being captured by other APCs to amplify the diffusion of MHC molecules, transmitting antigenic information to mucosal and systemic immune cells^46,47^. Recent studies have shown that exosomes isolated from *T. gondii* can induce humoral and cellular immune responses and control acute infection in mice^48^. In addition, experiments have shown that a novel cell-free vaccine composed of DC2.4 cell-derived exosomes can be transferred to the spleen and induce a Th1-mediated *T. gondii*-specific immune response in vivo, providing excellent anti-infection protection^49^.

However, exosome vaccines face many significant challenges, such as the lack of quality control, inconsistent purification standards, insufficient manufacturing, storage, management supervision, and poor biocompatibility. These problems must be overcome before they can be used for broad clinical applications and large-scale immunization programs^50^.

## Challenges in *T. gondii* vaccines development

**Fig. 2:**
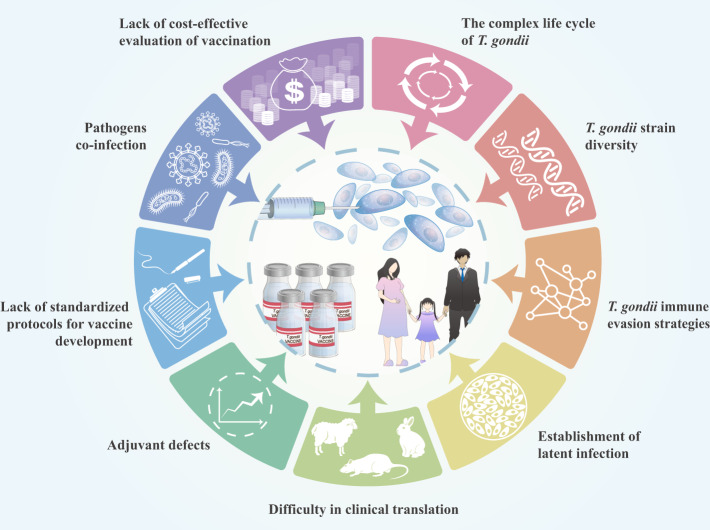
Challenges in *T. gondii* vaccine development.

Advances in the post-genomic era, such as bioinformatics, genomics, and proteomics, may assist in identifying and selecting new and effective antigens, thereby reducing future challenges^51^. However, future studies must address some limitations, such as adjuvants, methods, standardized immunization protocols, evaluation criteria, parasite strains, vaccines construction, and different animal models (Fig. 2).

### The complex life cycle of *T. gondii*

Due to a complex life cycle involving multiple hosts with diverse protein forms to express various invasion pathways, *T. gondii* exhibits high antigenic polymorphism and variability. Different states, such as tachyzoites, bradyzoites, or oocysts, exist in the host, and different antigens appear in different stages of *T. gondii*^52^. Consequently, vaccines should not be developed with a specific stage in mind. Using the above combination of antigens from various states is preferable, which is much more effective than a single antigen. An ideal immunogen vaccine should adequately encompass CD8^+^, CD4^+^, and B-cell epitopes from different stages of *T. gondii*^53^.

### *T. gondii* strain diversity

*T. gondii* strains firstly revealed three major lineages (Type I, II, and III), predominating in North America and Europe, 1995^54^. Although all *T. gondii* strains share a similar genetic background, their virulence and pathogenesis are strikingly different^54^. The type I strain is the most virulent and lethal in laboratory mice, infecting doses from a single living organism. The virulence of type II and type III was weaker than that of type I^55^. A fourth clonal lineage, known as type 12, has been found in North America, commonly in wildlife^56^. With the development of genetic analysis technology, a system to describe and interpret the evolutionary subdivision of *T. gondii* has been reported by examining thousands of isolates collected worldwide and genotyping them using three independent sets of polymorphic DNA markers. These cover 30 loci on all chromosomal and cytoplasmic genomes. The 138 unique genotypes revealed by the genetic diversity of markers in this system were grouped into 15 haplogroups that collectively define six major clades using cluster analysis^57^. Currently, unique genotypic strains recorded in the database ToxoDB (http://toxodb.org/toxo/) are up to ToxoDB#231. Due to the diversity of *T. gondii* strains, a vaccine based on one *T. gondii* strain may not be effective against other strains of different genotypes. The infection is not limited to a single strain. Therefore, the development of vaccines against most *T. gondii* strains is needed^58,59^. One stretagy could be finding the immunogenic conserved epitopes by analyzing the protein sequences of polymorphic antigens and the host immune responses to design a multi-allele vaccine that induce antibodies to the conserved epitopes and promote antibodies to multiple parasite strains^60^. Considering that this has been done for malaria, a similar approach can be applied for *T. gondii* vaccine development^60^. Finding the protein in *T. gondii* with limited antigenic diversity like apical membrane antigen 1 (AMA1) in *Plasmodium falciparum*. It means a vaccine including a small number of alleles might be sufficient to cover most naturally-circulating strains. It supports a multi-allele approach for developing polymorphic antigens for a *T. gondii* vaccine^61^.

### *T. gondii* immune evasion strategies

*T. gondii* invasion of host cells can interfere with the host’s immune system. *T. gondii* can avoid the damage of NO and hyperoxic substances by making the body’s high level of arginase competes with iNOS for the same substrate, reducing NO production^62^. *T. gondii* invasion-secreted inhibitor of STAT1 transcriptional activity (TgIST) translocates to the nucleus via the STAT signaling pathway, resulting in chromatin alteration and signal blocking^63,64^. *T. gondii* phosphorylates STAT3 and STAT6 by secreting ROP16 and upregulating M2 macrophage-like anti-inflammatory responses^65^. ROP5, ROP17, and ROP18 jointly prevent IFN-mediated IRGs from destroying the PVM^66^. GRA protects PV, helps *T. gondii* survive in host cells, enhances *T. gondii* virulence, and increases tissue cysts^67–69^. In addition, *T. gondii* effectors inhibit nuclear factor-κB (NF-κB) function by interfering with Enhancer of Zeste 2 Polycomb Repressive Complex 2 Subunit (EZH2) or tripartite motif-containing 21 (TRIM21)^70,71^. As a pro-inflammatory molecule, macrophage migration inhibitory factor (MIF) is a crucial mediator of immune responses against various pathogens, including parasites^72^. *T. gondii* MIF directly binds to human macrophage MIF receptor CD74 to prevent macrophage apoptosis from obtaining sufficient nutrients and time to reproduce by using this “tent” to disseminate without being killed by the host immune system^73^. *T. gondii* successfully evades immune surveillance by manipulating the host immune system through various mechanisms, which has challenged effective vaccine development. However, until now, the interaction between *T. gondii* and its host must be further studied. Screening the whole *T. gondii* genome for molecules that may play a key role in immune escape or pathogenesis and further functional identification is critical for the in-depth study of *T. gondii* genetics, immune escape, and vaccine development.

### Establishment of latent infection

When rapidly proliferating *T. gondii* tachyzoites differentiate into cyst-forming bradyzoites, the parasitic vacuole in which *T. gondii* replicates is remodeled into a highly glycosylated cyst wall. *T. gondii* can prolong the infectious period by establishing a latent or chronic state, avoiding immune clearance by slow replication, altering immunogenicity, and reducing the impact on the host. This continuous phase, which can reoccur or contribute to disease transmission, impedes curing and eradicating infectious diseases^74^. Recently, a Myb-like transcription factor (BFD1) was identified as required for bradyzoite differentiation in *T. gondii*^75^. BFD1 accumulates during stress, and its synthetic expression is sufficient to drive differentiation, incorporating promoters of many stage-specific genes. BFD1 provides a genetic switch for studying and controlling *T. gondii* differentiation and will inform the prevention and treatment of chronic infections^75^. Therefore, in studying the *T. gondii* vaccines, we should fully consider how to use the key molecule BFD1 to enhance the immune protective effect of the vaccines.

### Difficulty in clinical translation

Laboratory mice are considered the most widely used model for studying the pathogenesis and immunological events involved in controlling or preventing *T. gondii* infection and have provided important insights for developing vaccines against toxoplasmosis. However, the transfer of experimental studies to clinical practice has been hampered mainly by species differences between experimental mice and humans. First, IRGs, a class of proteins involved in the mice’s early immune response, can aggregate on PVM to form holes, destroying the replication region of *T. gondii*^76^. Humans do not have IRG resistance systems^77^. Second, profilin is an actin-modifying protein that binds and activates TLR11 and TLR12, promotes IL-12 production, inducing NK cells to produce IFN-γ, which plays an important role in eliminating *T. gondii* tachyzoites^78^. However, TLR12 is not present in humans, and TLR11 is also a functional pseudogene^79^. *T. gondii* antigen can only be presented by ordinary antigen-presenting cells to activate cellular immunity against infection. As a result, antigens found in mice may not protect humans or other animals. Therefore, it is very inappropriate to directly transfer the experimental results obtained using mice as the research object to clinical practice.

Transgenic mice expressing MHC class I and II of human origin have emerged as ideal models for *T. gondii* vaccine development, which has the potential to facilitate the development of human vaccines^33^. HLA transgenic mouse model has been widely used in preclinical and experimental studies. However, MHC genes are highly polymorphic in humans and mammals. Furthermore, it has significant geographical and ethnic differences in the distribution of its dominant genotypes. In addition, most of the common international MHC transgenic mouse models are HLA-I or HLA-II single-transgenic mice, which still cannot examine the synergistic immunomodulatory effects of HLA-I and HLA-II class molecules in the organism. Therefore, there is a pressing need to construct HLA-I/II double transgenic mouse models that cover as much of the population as possible with HLA genetic traits, which is a difficult task.

### Adjuvant defects

Currently, *T. gondii* vaccines have evolved from live attenuated vaccines to subunit and genetically engineered vaccines, most of which are weakly immunogenic. As a result, some adjuvants are required to deliver the vaccine and elicit a robust immune response. Adjuvants can reduce antigen dose, improve vaccine efficacy in immunocompromised individuals, and enhance the immune response to protect the vaccines against highly mutagenic pathogens. Currently, adjuvants such as aluminium salts, Toll-like receptor agonists, liposomes, immunostimulating complexes (ISCOM), and CPG motifs synergistically induce a potent immune response^80^. However, there are some emerging issues with the addition of adjuvants. For example, the uniformity of aluminium salt adjuvant particles is poorly controlled and easily absorbed by other nonimmune cells, resulting in poor immune protection. Toll-like receptor agonist adjuvants help pathogens evade the host’s immune system. Recent experiments have shown that the adjuvant activity of Al-PRP mixtures is stronger than that of single adjuvants (alum or PRP)^81^. Accordingly, combining adjuvants could be considered to overcome the disadvantages of using different adjuvants alone.

### Lack of standardized protocols for vaccines development

It is difficult to compare the efficacy of different candidate vaccines due to the different strains, life cycle stages of *T. gondii* used in different inoculation routes and doses, and animal models. Different laboratories are not easily comparable. Therefore, establishing standardized protocols for vaccine development is urgently required.

### Pathogens co-infection

When humans or other hosts are exposed to the natural environment, infections of multiple pathogens can occur. *T. gondii* infection induces a Th1-type immune response, while other pathogens, such as the helminth, may induce Th2 immune responses^82^. This mixed infection also poses a significant challenge for *T. gondii* vaccine development, which researchers must assess.

### Lack of cost-effective evaluation of vaccination

The only commercial vaccine is the attenuated tachyzoite S48 strain (Toxovax®) used in sheep. It is produced to be supplied as a concentrated suspension of tachyzoites and diluent. This vaccinated sheep can reduce abortion and neonatal mortality and improve the birth weight of lambs. Toxovax® vaccine is only used in the UK, New Zealand, France, and Ireland^83^. However, because it is based on tachyzoite growth in mammalian cell cultures, this vaccine has a very short shelf life and high production costs. Concerns have also been raised about its safety, as live attenuated vaccines risk reverting to wild strains and infecting humans^7^. In addition, no cost-effectiveness has been reported for this vaccination program, explaining why this vaccine has not been implemented in other countries^84^. Many different pharmacological treatments are available for *T. gondii* infection, and farmers may have no incentive to spend money on this vaccine if there is no obvious economic return^85^.

Another method is the strategic vaccination of cats to reduce environmental contamination by reducing oocyst expulsion. Several studies on *T. gondii* vaccines for cats have also been conducted^86^. The T-263 strain completely prevents oocyst-shedding in the tested animals^8^. Recently, Ramakrishnan et al. modified a *T. gondii* strain with defective fertilization, reduced fecundity, and the inability to produce normal oocysts. Immunization of cats with this engineered strain completely prevented oocyst excretion following *T. gondii* infection. These results are very encouraging^10^. A vaccination program with an adequate cost-benefit analysis would help guide vaccine development and market application^87^. However, models and conclusions regarding cost-effectiveness analyses of *T. gondii* vaccination programs for cats have been suboptimal. Sykes et al. created a mathematical model that can be used to predict average vaccination levels in domestic cat populations. They found a critical vaccine cost threshold above which no one would use the vaccine. Vaccine costs slightly below this threshold would result in higher vaccine use, significantly reducing the seropositivity rate in the domestic cat population. Unfortunately, domestic cats can only achieve herd immunity at no vaccine cost^88^. Another model study showed that the prospects for preventing human toxoplasmosis caused by oocysts by vaccination in large cat populations are not promising due to the extensive vaccination coverage required. This vaccination method is only effective in small cat populations such as those on farms^89^.

*T. gondii* infection is common in warm-blooded animals, which provides a constant threat to humans and livestock. Eradication of *T. gondii* infection is almost impossible due to the wide range of natural reservoirs. In the case of *T. gondii* infection, a vaccine would be a complement to treatment and other preventive measures rather than a substitute. It is also costly for a parasite that cannot be eradicated and will only clinically affect a small number of people^90^. To be cost/effective, a vaccination program should prove cheaper than treatment. In addition, a vaccine targeting high-risk groups may be valuable, for example, women of childbearing age or HIV-positive patients. Therefore, it is also important to conduct adequate cost-effectiveness studies of *T. gondii* vaccination programs for this specific population, either globally or in different countries or regions, in conjunction with experts in economics and statistics.

Current *T. gondii* vaccine research indicates that live attenuated vaccines are the most effective option. Live attenuated oral vaccines can mimic natural infections. We believe it will be the most promising vaccine if they overcome the abovementioned challenges, especially if the vaccine can overcome their unknown genetic background. Besides, with the wide use of CRISPR technology, generating gene deletion mutants as live vaccines has become feasible and provides a novel approach for controlling toxoplasmosis.

## Conclusion

To summarize, *T*. *gondii* is an obligate intracellular parasite with global distribution and important medical and veterinary implications. With the identification of neoantigens, adjuvants, and immunization strategies, significant progress has been made in developing *T. gondii* vaccines. Candidate vaccines include recombinant antigens, multi-epitope antigens, DNA or RNA, and microparticles. The advantages of these vaccines have been extensively studied in various animal models to assess their potential to elicit cellular and humoral immune responses and protect against the *T. gondii* challenge. Next, a careful selection of highly immunogenic antigens covering different stages of the *T. gondii* life cycle should be performed to construct a multi-antigen vaccine, combined with appropriate adjuvants and delivery systems, using HLA transgenic mice covering populations with predominant HLA genotypes for vaccines protection evaluation. Furthermore, adequate, cost-effective studies for *T. gondii* vaccination programs should be conducted, particularly for women of childbearing age and HIV-positive patients. Although there are many challenges in developing a *T. gondii* vaccine, we remain optimistic that developing an effective vaccine to prevent and treat toxoplasmosis remains possible.

## Data Availability

Data sharing is not applicable to this article as no datasets were generated or analyzed during the current study.

## Code availability

Any custom code or mathematical algorithm is not applicable to this article.
